# Inhibition of *Candida parapsilosis* Fatty Acid Synthase (Fas2) Induces Mitochondrial Cell Death in Serum

**DOI:** 10.1371/journal.ppat.1002879

**Published:** 2012-08-30

**Authors:** Long Nam Nguyen, Gabriele Vargas Cesar, Giang Thi Thu Le, David L. Silver, Leonardo Nimrichter, Joshua D. Nosanchuk

**Affiliations:** 1 Department of Medicine (Division of Infectious Diseases), Albert Einstein College of Medicine, New York, New York, United States of America; 2 Signature Research Program in Cardiovascular & Metabolic Disorders, DUKE-NUS Graduate Medical School, Singapore; 3 Laboratório de Estudos Integrados em Bioquímica Microbiana, Instituto de Microbiologia Professor Paulo de Góes, Universidade Federal do Rio de Janeiro, Rio de Janeiro, Brazil; 4 University of Hamburg, Biocenter Klein Flottbek, Department of Molecular Phytopathology and Genetics, Hamburg, Germany; 5 Department of Microbiology and Immunology, Albert Einstein College of Medicine, New York, New York, United States of America; University of Toronto, Canada

## Abstract

We have recently observed that a fatty acid auxotrophic mutant (fatty acid synthase, *Fas2Δ/Δ*) of the emerging human pathogenic yeast *Candida parapsilosis* dies after incubation in various media including serum. In the present study we describe the mechanism for cell death induced by serum and glucose containing media. We show that *Fas2Δ/Δ* yeast cells are profoundly susceptible to glucose leading us to propose that yeast cells lacking fatty acids exhibit uncontrolled metabolism in response to glucose. We demonstrate that incubation of *Fas2Δ/Δ* yeast cells with serum leads to cell death, and this process can be prevented with inhibition of protein or DNA synthesis, indicating that newly synthesized cellular components are detrimental to the mutant cells. Furthermore, we have found that cell death is mediated by mitochondria. Suppression of electron transport enzymes using inhibitors such as cyanide or azide prevents ROS overproduction and *Fas2Δ/Δ* yeast cell death. Additionally, deletion of mitochondrial DNA, which encodes several subunits for enzymes of the electron transport chain, significantly reduces serum-induced *Fas2Δ/Δ* yeast cell death. Therefore, our results show that serum and glucose media induce *Fas2Δ/Δ* yeast cell death by triggering unbalanced metabolism, which is regulated by mitochondria. To our knowledge, this is the first study to critically define a link between cytosolic fatty acid synthesis and mitochondrial function in response to serum stress in *C. parapsilosis*.

## Author Summary

*Candida parapsilosis* is a human opportunistic pathogen associated with significant morbidity and mortality, especially in immunocompromised individuals such as premature, low-birthweight neonates. Our prior studies have indicated that *C. parapsilosis* effectively utilizes fatty acids/lipids for growth and virulence. We now show that inhibition of the fatty acid synthase (Fas2) results in a hypersensitivity to serum, indicating that yeast cell survival and replication in serum medium or in vivo is dependent on Fas2. Serum hypersensitivity of Fas2-inhibited yeast cells is due to mitochondrial mediated dysregulation of metabolism. Thus, we conclude that Fas2 is candidate antifungal target to combat disseminated fungal infections.

## Introduction

Fatty acid biosynthesis plays a significant role in the growth and survival of diverse organisms. In yeasts, the de novo fatty acid synthesis pathway produces and regulates essential fatty acid species such as saturated (SFA) and unsaturated (UFA) fatty acids that are required for generation and maintenance of cell membranes. Inhibition of enzymes in this pathway, such as fatty acid synthase and fatty acid desaturase, impedes yeast cell growth unless appropriate exogenous fatty acids are provided [1]–[3]. Thus, inhibition of a single enzyme in the *de novo* fatty acid synthesis pathway can result in profoundly altered physiological phenotypes and may impact virulence in pathogenic yeasts.

Fatty acid synthesis pathways have been considered as targets to combat bacterial infection. For example isoniazid is a fatty acid synthesis inhibitor that is used to treat tuberculosis [4], [5]. Platensimycin, a specific inhibitor of bacterial beta-ketoacyl-acyl-carrier-protein synthase I/II (FabF/B), is in a clinical trial for resistant strains of *Staphylococcus aureus* [6], [7]. Targeting bacterial fatty acid synthesis pathways has recently been criticized after a study showed that Gram-positive fatty acid synthesis deletion mutants could overcome the lack of de novo fatty acid synthesis using exogenous fatty acids from serum [8]. However, another study has shown that *S. aureus* FASII is essential [9]. Although, the potential of exploiting the fatty acid biosynthesis pathway for targeting microbial infections is still in debate, these studies suggest the importance of evaluating the efficacy of drugs in more complex media such as serum.

*Candida* species are the 4^th^ most common isolates in blood cultures. Hence, survival in serum is key to pathogenesis. There is limited information regarding targeting fatty acid synthesis in human pathogenic fungi. However, inhibition of calcineurin or threonine biosynthesis in *Candida albicans* induces cell death after serum treatment, suggesting that these pathways could be ideal for antifungal drug development [10], [11]. Notably, serum induces virulence traits such as filamentation and biofilm formation in *Candida* species [12]. Antifungal drug efficacy is also reduced in serum compared with other media [13]–[15], increasing the difficulty for treatment of systemic infections. *Candida parapsilosis* has emerged as an important human pathogen and it is currently the second most common *Candida* species globally [16], [17]. Risk for infection is especially high in immunocompromised patients and low-birthweight, premature neonates. The fungus exhibits many clinical features in common with other *Candida* species, such as an ability to cause systemic infections or superficial infections, and drug resistance. However, little is known about the pathobiology of *C. parapsilosis*. We have recently demonstrated that *C. parapsilosis* fatty acid synthase (Fas2) is essential for viability in the absence of exogenous fatty acids and *FAS2* disruptants (*Fas2Δ/Δ*) have attenuated virulence in mice [3], [18]. Additionally, *C. parapsilosis Fas2Δ/Δ* yeast cells are hypersensitive to serum. Moreover, blocking of Fas2 in wild-type (WT) *C. parapsilosis* using the inhibitor cerulenin induces cell death in serum [3]. Our findings highlight the potential importance for developing novel drugs that enhance the susceptibility of yeast cells in serum. In the current study, we investigate the mechanism of cell death induced by serum and glucose containing media in *C. parapsilosis Fas2Δ/Δ* yeast. Here, we report that inhibition of Fas2 leads to the mitochondria-dependent cell death of *C. parapsilosis Fas2Δ/Δ* yeast in response to glucose in the media.

## Materials and Methods

### Strains and culture conditions

*C. parapsilosis* strains used in this study are: wild-type GA1 (WT) [19], *Fas2Δ/Δ* and the reconstituted strain (*Fas2Δ/FAS2*) [3]. Mitochondrial DNA (mtDNA) disrupted *C. parapsilosis Fas2Δ/Δ* (*Fas2*-*rho^−^*) yeasts were generated in this study as follows. *Fas2Δ/Δ* yeast cells were grown overnight in YPD supplemented with saturated fatty acids as described [3]. The yeast cells were plated in YPDT40 containing 10 µg/ml ethidium bromide [20]–[22]. The plates were incubated at 30°C for 4 days. Small yeast colonies were picked and grown in YPD supplemented with saturated fatty acids. To identify yeast strains in which mtDNA was deleted partially or completely, the yeast cells were cultivated in defined YNB medium without addition of glucose but supplemented with 2% glycerol as the sole carbon source and saturated fatty acids. Since a lack of mtDNA suppresses yeast cell growth in non-fermentable carbon sources such as glycerol, yeast cells exhibiting severe growth defects in this condition were selected. The mtDNA content in the selected yeasts were assayed using specific primers for subunit I of cytochrome c oxidase (Cox1, COX1F: CTTATTTACTATTGGTGGTTTAACAGG and COX1R: TCAGAGTAAGTATGATATTGTGCTGG). A primer pair specific for nuclear actin gene was used for control (ACT1F: GAACAAGAAATGCAAACCTCA and ACT1R: GAACCACCAATCCAGACAGA).

*C. parapsilosis* strains were maintained at −80°C in 35% glycerol. Yeast cultures were incubated in an orbital shaker set at 150 rpm and 30°C. If not otherwise mentioned, the strains were grown in either YPD (1% yeast extract, 2% bactopeptone, 1% glucose) or YPDT40 (YPD plus 1% Tween 40, and 0.01% palmitic and myristic acids, pH 6.5). For serum, yeast cells were incubated in 50% heat-inactivated fetal bovine serum diluted with sterile water. To assess the effects of serum components, serum was fractionated using membrane cut-off columns (Millipore) based on molecular weights. The eluates and retention fractions were adjusted to the initial volume of serum with PBS. Lipids were extracted from serum with chloroform for 1 hour with vigorous shaking. The organic phase was collected by centrifugation and evaporated under nitrogen gas. Lipids were then dissolved in 1% ethanol and then with PBS (Final ethanol concentration in lipid extracts was less than 0.01%). YPD, yeast extracts, peptone, YNB, YNB supplemented with amino acids were all purchased from Difco (USA).

### Survival assays

Initially, WT and *Fas2Δ/Δ* yeast cells were grown in liquid YPD or YPDT40, respectively. Yeast cells were cultured overnight, collected by centrifugation, washed three times, and then diluted with PBS and counted using a hemocytometer. For all the growth and survival assays, concentrations of 2×10^6^/ml yeast cells were incubated in 5 ml of the different mediums: serum fractions, YPD, YPD components, YPD supplemented with 0.01% palmitic acid (C16:0), YNB components as described in the text, or YNB supplemented with 1% glucose, glycerol, ethanol, 2-deoxy glucose or 0.01% palmitic acid. Water was used as control. For the spot assays, 50 µls (approximately 10^5^ cells) of above-mentioned cultures were subjected to series of five-fold dilution in PBS and 2.5 µls of the initial cultures and respective dilutions were spotted on YPD plates for the WT and YPDT40 for the mutant strains. The plates were incubated at 30°C for two days before being photographed. For measurement of cell density, yeast growth was monitored using a microtiter reader (Labsystem Multiskan MS).

Similarly, cell death was also assessed using flow cytometry (FACS). Yeast cells inoculated with 50% serum in the presence or absence of 50 and 75 mM hydroxyurea, or with or without 50 µg/ml cycloheximide, or 20 and 50 µg/ml nocodazole, or 20 and 40 µM antioxidant N-acetyl cysteine, or YNB supplemented with or without 1% glucose, ethanol, or glycerol as the sole carbon source were stained with 2.5 mM Sytoxgreen (Invitrogen) at room temperature for 1 hour and then subjected to FACS analysis. Negative and positive green fluorescence yeast cells were recorded with a MacsQuant flow cytometer. The percentages of negative and positive green fluorescent cells were evaluated from >25,000 cells.

### Drug inhibition assays

For all the experiments, yeast cells grown overnight were washed three times and suspended in PBS. Aliquots of 2×10^6^ cells/ml were inoculated in 50% serum or YNB with glucose media with or without one of the following drugs: 10 µg/ml rotenone, 2.5 µg/ml antimycin A, 1 mM cyanide (NaCN), 2.5 mM azide (NaN_3_), 10 µg/ml oligomycin, 1 mM carbonyl cyanide 4-(trifluoromethoxy)phenylhydrazone (FCCP), 50 µg/ml cycloheximide (CHX), 75 mM hydroxyurea, 100 µM wortmannin, 1.38 mM fumonisin b1, or 10 µg/ml cerulenin (negative control), which were purchased from Sigma. The cultures were incubated at 30°C with orbital shaking. Aliquots of the initial cultures were plated on YPDT40 for enumeration. The percent inhibition was the number of viable yeast cells recovered from drug inhibition compared with controls (without drug) and expressed by percentage.

### Murine infection

A/J mice (female, 6–8 weeks of age; National Cancer Institute) were infected intravenously with 3×10^7^ WT or *Fas2Δ/Δ* yeast cells in 100 µl PBS. Mice were sacrificed at 3 or 5 days after infection. Livers, kidneys, and spleens were removed and organ homogenates were plated on appropriate agars for enumeration of CFUs. For histological examinations, kidneys of infected A/J mice were removed at day 5 after infection and fixed with formalin. The kidneys were embedded in paraffin, sectioned, and stained with periodic acid-Schiff (PAS) stain. Renal sections were examined using an Olympus AX70 microscope (Olympus America Inc.) with a 40× objective.

### Ethics statement

Animal experiments were performed according to the Guide published by the Institute of Laboratory Animal Resources of National Research Council. Animal care for this study was approved by the institutional Animal Care and Use Committee of Albert Einstein College of Medicine under the protocol number 20080604.

### Microscopy studies

To study the production of daughter cells after serum or YNB (with glucose) incubation, yeast cells were labeled with 40 µg/ml NHS-Rho (Pierce) in PBS for 1 hour at 4°C before incubation. Yeast cells were then collected at intervals and stained with 10 µM Sytoxgreen for 30 min to evaluate the viability. To investigate the role of the production of reactive oxygen species (ROS), yeast cells were inoculated with 50% serum or YNB (with glucose) in the presence or absence of 1 mM NaCN or 2.5 mM NaN_3_. The cells then were collected at intervals and co-stained with 10 µM Sytoxgreen and 5 µg/ml dihydrorhodamine (DHR) 123 (Sigma) in PBS for 30 min at room temperature (RT). Mitochondrial morphology was investigated by staining yeast cells incubated with serum or YNB (with glucose) in the presence or absence of 1 mM NaCN or 2.5 mM NaN_3_ with 10 nM mitrotracker red CM-H_2_XRos (Invitrogen) in PBS for 30 min at RT. Serum or YNB (with glucose) treated yeast cells were stained with 10 µM FUN-1 (Invitrogen) to study metabolic activity. Yeast cell viability was assayed by staining the cells with 10 µM Sytoxgreen or 20 µg/ml propidium iodine (PI, Sigma) in PBS for 30 min at RT. All assays were analyzed on an Axiovert 200 M microscope (Carl Zeiss Inc.) with appropriate fluorescence filters and the images were digitally captured with an AxiocamMR.

For electron microscopy, *C. parapsilosis* yeast cells cultivated with or without NaCN were fixed in 2.5% (v/v) glutaraldehyde, 4% formaldehyde and 10 mM CaCl_2_ in a 0.1 M sodium cacodylate buffer, post-fixed in 1% (w/v) OsO_4_ plus 0.8% potassium ferrocyanide, dehydrated in acetone series, and embedded in Spurr resin. Sections were stained for 30 min in uranyl acetate, 5 min in lead citrate, and observed in a JEOL 1200EX electron microscope (JEOL, Akishima, Tokyo) operating at 80 kV.

### Cell death assays

Analysis of cell death markers were performed using Annexin V and PI with yeast protoplasts as described [23], [24] with minor modifications. Briefly, yeast cells grown in YPD or YPDT40 overnight, washed and incubated in 50% serum. The yeast cells were then collected by centrifugation and washed in 0.5 ml washing buffer (50 mM K_2_HPO_4_/5 mM EDTA/50 mM DTT (pH 7.2) for 30 min at 30°C. The cells were then digested with a mixture of cell wall degrading enzymes (Chitinase, beta-glucuronidase, zymolyase; Sigma) prepared in 50 mM KH_2_PO_4_, 40 mM 2-mercaptoethanol, and 2.4 M sorbitol for 45 min at 30°C. Protoplasts were collected by centrifugation and stained with 20 µg/ml FITC-Annexin V (BD Biosciences) and 20 µg/ml PI in binding buffer (10 mM Hepes/NaOH, 40 mM NaCl, 50 mM CaCl_2_, 1.2 M sorbitol, pH 7.4/) for 15 min at RT. Cells were mounted in microscope slides and visualized by microscopy as described above. The cells labeled with Annexin V or PI were counted and expressed as percents of the total cells in the visualized fields.

### DNA content analysis

Yeast cells inoculated with 50% serum as described in survival assays in the presence or absence of 75 mM hydroxyurea were collected at 1 hour intervals and immediately fixed with 70% ethanol for 1 hour at room temperature. Yeast cells were washed with PBS and treated with 50 µg/ml RNase in 0.05 M sodium citrate, pH 7.2 for 3 hours at 37°C followed by overnight incubation with 50 µg/ml proteinase K in 0.05 M Tris-HCl, 0.01 M CaCl_2_, pH 8 at 55°C. Yeast cells were then stained with 5 µM Sytoxgreen in 0.05 M sodium citrate, pH 7.2 for 15 min. Stained cells were analyzed and sorted using a MacsQuant flow cytometer. Green fluorescence signals were recorded and histograms were generated. The percentage of G1 populations (2 N, diploid cells) and G2 populations (4 N, tetraploid cells) were calculated from 30,000 cells after gating to exclude unstained and clumped cells.

### Statistical analysis

The significance of differences between sets of data was determined by Newman-Keuls using Graph Prism version 5.02 for Windows (California, USA). P values<0.05 were considered significant.

## Results

### *Fas2Δ/Δ* strain hypersensitivity

**Figure 1:**
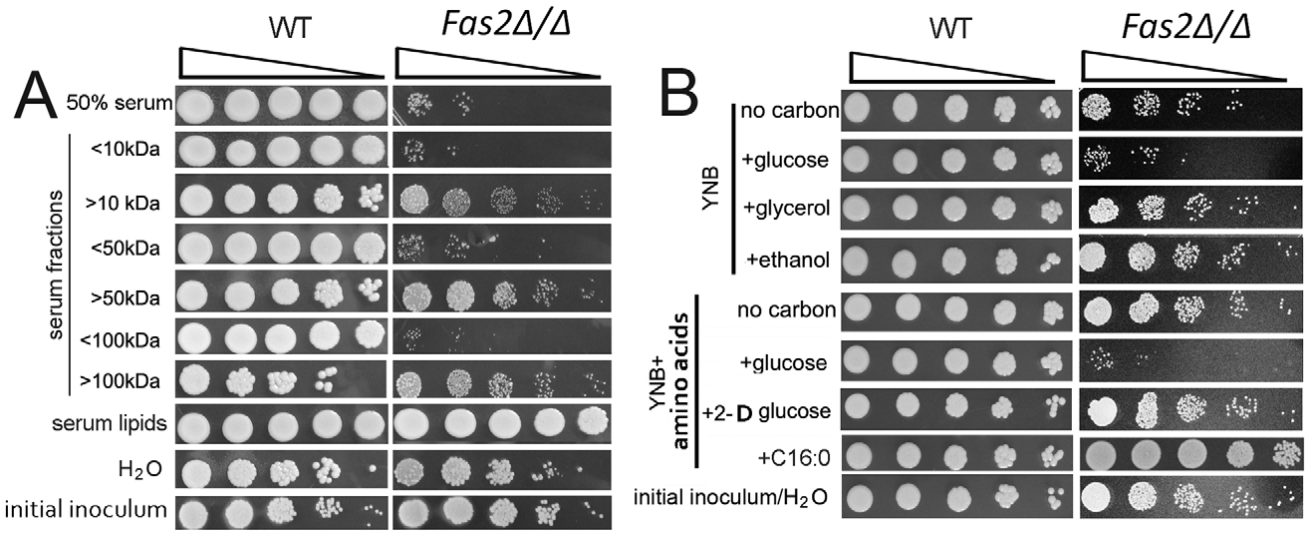
*Fas2Δ/Δ* strain is susceptible to serum and glucose containing media. A, Growth of wild-type (WT) and survival of *Fas2Δ/Δ* yeast cells in the indicated serum fractions and lipid extracts from serum. Aliquots from 24 hour cultures were serially diluted and WT were plated on YPD whereas the *Fas2Δ/Δ* were inoculated on YPDT40. Relative to the *Fas2Δ/Δ* grown in YPDT40 or to WT yeast cells in the different fractions, the survival of *Fas2Δ/Δ* yeast cell growth was impeded in lower serum fractions, but not in upper serum fractions. WT and *Fas2Δ/Δ* yeast cells grew similarly in medium supplemented with serum lipids. H_2_O was used as control. “<” and “>” kDa indicate the lower and upper serum fractions, respectively, eluted from corresponding membrane cut-off columns. B, Viability of *Fas2Δ/Δ* yeast cells yeast cells after 24 hours of incubation in YNB with or without amino acids supplemented with 1% of the indicated carbon source. The survival of *Fas2Δ/Δ* yeast cells was reduced in the presence of glucose in the media. YPD with glycerol, amino acids, 2-D-glucose, or palmitic acid (C16:O) did not induce *Fas2Δ/Δ* yeast cell death. Experiments were performed at least three different times and similar results were obtained.

We previously observed that the *Fas2Δ/Δ* strain was hypersensitive to serum. To gain insight into the serum factors that were toxic to *Fas2Δ/Δ* yeast cells, we tested the survival of yeast cells in different serum components. We first determined that serum, heat-denatured serum, and proteinase K treated serum were equally toxic to the *Fas2Δ/Δ* yeast cells (data not shown; [10], [11]). Next, we fractionated the serum by molecular weight and found reduced viability of the mutant yeast cells in the <10 kDa fraction (Figure 1A). As they contained the <10 kDa fraction, the <50 and <100 kDa fractions similarly sensitized the *Fas2Δ/Δ* yeast cells. In contrast, serum fractions of >10, 50 or 100 kDa were not inhibitory; however, the addition of glucose to these fractions efficiently killed the Fas2 mutant cells (Figure S1). WT yeast cells grew in all the conditions tested, although the growth of WT in the >100 kDa fraction was lower compared to the other fractions (Figure 1A). The *Fas2Δ/Δ* yeast cells were able to grow in lipids isolated from serum, indicating that serum lipids support mutant growth in non-serum media (Figure 1A). We also observed that incubation of *Fas2Δ/Δ* yeast cells in water did not significantly decrease viability. These data indicated that serum factors <10 kDa were responsible for the *Fas2Δ/Δ* strain susceptibility.

To better identify toxic factors, we assessed the impact of additional factors that are present in serum. We determined the rates of cell death in YNB media with or without amino acids and found that the addition of amino acids to media did not accelerate *Fas2Δ/Δ* yeast cell death (Figure 1B). Additionally, we found that the *Fas2Δ/Δ* yeast cells were not sensitive to YNB (with amino acids) supplemented with diverse ions or vitamins such as niacin (data not shown; [10], [11]). Interestingly, we found that the mutant yeast cells rapidly died when 1% glucose was added to YNB media regardless of the presence or absence of amino acids (Figure 1B). A serum glucose concentration of ∼0.1% was similarly toxic to the mutant yeast cells (Figure S1B). These results demonstrated the *Fas2Δ/Δ* yeast cells had decreased survival when glucose was present in the media. To further demonstrate that glucose catabolism resulted in toxicity, we replaced glucose with 2-deoxy glucose, which can be taken up by yeast, but is not a substrate for glycolysis. YNB (with amino acids) supplemented with 1% 2-deoxy glucose did not toxify *Fas2Δ/Δ* yeast cells (Figure 1B). Furthermore, the addition of non-fermentable carbon sources, such as glycerol or ethanol, to YNB did not induce death of the mutant yeast cells (Figure 1B, S1). The mutant yeast cells were viable in water and grew in YNB supplemented with palmitic acid (Figure 1B). These data indicated that the glucose in YNB medium was a key factor in *Fas2Δ/Δ* yeast cell hypersensitivity, and cell death was not due to starvation.

### *Fas2Δ/Δ* yeast cells are dysregulated in serum and glucose containing medium

**Figure 2:**
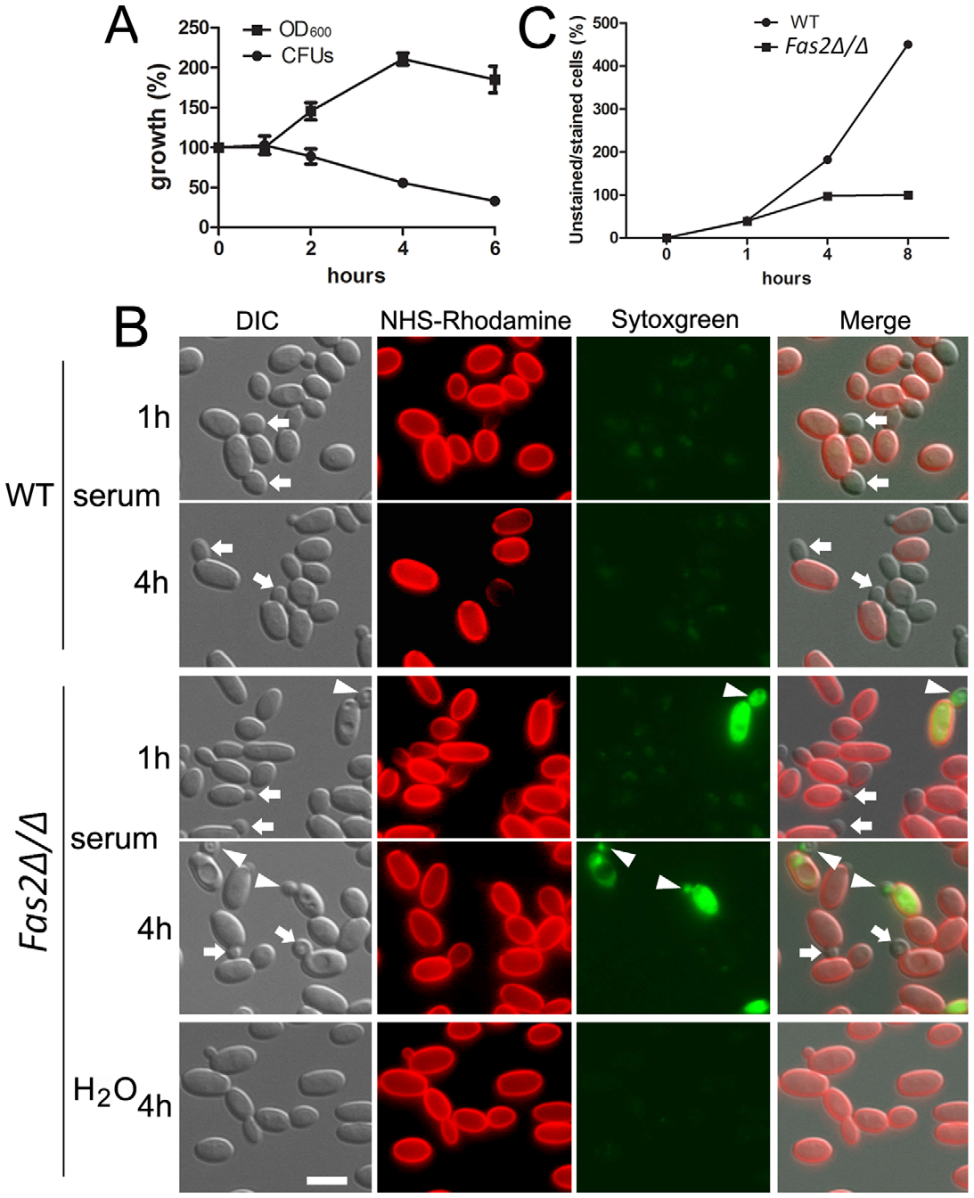
Serum incubation results in dysfunction in bud formation in *Fas2Δ/Δ* yeast cells. A, *Fas2Δ/Δ* yeast cell growth in serum as measured by cell density compared with plating for CFUs demonstrates that the mutant yeast generate daughter cells, but the cells within the culture begin to die concomitantly. B, *Fas2Δ/Δ* yeast cells were able to produce one progeny, but the daughter cells were not released. Arrows show new unstained buds. Arrowheads indicate unlabeled buds on NHS-Rhodamine labeled *Fas2Δ/Δ* parental cells (initial inoculum) where both the parental cell and bud are labeled with sytoxgreen, an indicator of death. Scale bar: 5 µm. C, Number of new yeast cells produced by WT and *Fas2Δ/Δ* after serum incubation. Experiments were performed at least twice with similar results.

The *Fas2Δ/Δ* yeast cells grew in YPD supplemented with saturated fatty acids [3] and in medium supplemented with lipids extracted from serum. Therefore, we reasoned that the mutant yeast cells might be able to initiate growth in serum before dying. Indeed, we observed that the cell density (OD_600_) of the *Fas2Δ/Δ* yeast cell cultures in serum increased by approximately 100% after 4 h and remained constant thereafter (Figure 2A). In contrast, we did not observe increased CFUs by plating methods for the same period. To clarify the differences between OD and CFU assays, we examined the mutant yeast cells microscopically. Before inoculation into serum, the log-phase yeast cells were stained with NHS-Rhodamine to differentiate the initial inoculum from their progeny. The NHS-Rhodamine is bound to the cell wall of the yeasts in the initial inoculum and is not transferred to the daughter cell, in which cell wall synthesis occurs *de novo*, making it useful in tracking the fate of the parent inoculum. The WT yeast cells produced daughter cells after 1 h of serum incubation, whereas small buds arose from the *Fas2Δ/Δ* yeast cells in the same period (Figure 2B). WT *C. parapsilosis* doubling time is about 1 h in standard media [25]. In our present work, we observed a slight reduction in WT yeast cells replication as the culture population was only 40% newly formed cells after 1 h serum incubation, which might indicate an inhibitory effect of NHS-Rhodamine or, more likely, serum on proliferation. In the same conditions, we observed that 40% of *Fas2Δ/Δ* yeast cells produced small buds after 1 h, but the CFUs did not increase until 4 h, which correlated with the OD measurements (Figure 2A, C). Interestingly, we observed that new small daughter cells of the mutant were not able to bud off from the mother cells (red cell wall). The results initially suggested that *Fas2Δ/Δ* yeast cells failed to complete the cell division cycle and it seemed that the mother cells were halted at G2/M stage after 4 hours. In the same period, WT yeast cells produced new progenies (Figure 2B). Intriguingly, we observed that both newly emerged buds and mother cells of the mutant died after 1 h of incubation (Figure 2B). Similar results with the *Fas2Δ/Δ* strain were also observed with YNB supplemented with 1% glucose (data not shown). Incubation of the mutant cells in water did result in the production of daughter cells and there were no changes in viability. Taken together, the results demonstrated that *Fas2Δ/Δ* yeast cells were able to generate a single un-released progeny in media with glucose prior to expiring, indicating that the cell cycle was not arrested in nutrient-rich media and that perhaps glucose triggered active cellular processes leading to cell death.

### Inhibition of Fas2 did not suppress cell cycle progression of yeast cells

**Figure 3:**
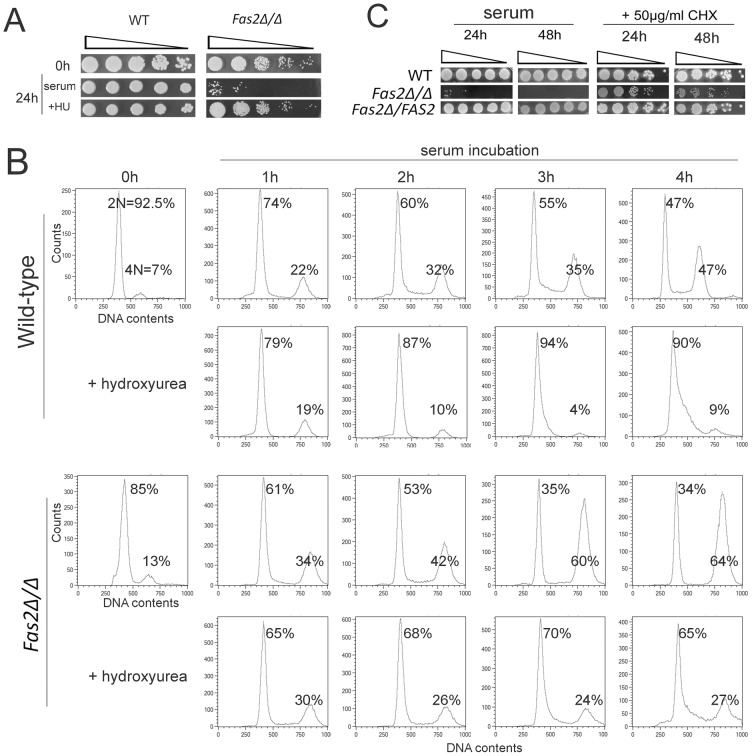
*Fas2Δ/Δ* yeast cells did not arrest cell cycle progression upon serum incubation. A, The survival of *Fas2Δ/Δ* yeast cells in serum was enhanced by 75 mM hydroxyurea (HU). The yeast cells before (0 h) and after (24 h) serum incubation were serially diluted and spotted on YPD media for WT and YPDT40 for *Fas2Δ/Δ* strain. B, DNA content of the WT and *Fas2Δ/Δ* strains before (0 h) and after 1–4 h of serum incubation with the presence or absence of 75 mM hydroxyurea was measured by FACS. Cultures were collected at indicated intervals and processed for DNA content analysis by staining with Sytoxgreen. The percentages of yeast cells at G1 and G2 stage were calculated. C, Cycloheximide (CHX) rescued *C. parapsilosis* FAS2 mutant cells from serum induced death. In contrast, inhibition of protein synthesis by CHX slightly reduced WT and reconstituted (*Fas2Δ/FAS2*) *C. parapsilosis* yeast cell growth. Experiments were performed twice with similar results.

The mutant yeast cells failed to divide/bud after 4 h of incubation in serum. Thus, we hypothesized that inhibition of Fas2 resulted in a lack of fatty acids/lipids for membrane synthesis in new daughter cells, but synthesis of other components were still occurring, resulting in an asymmetry of cellular contents. We explored this further by examining the synthesis of DNA in the *Fas2Δ/Δ* strain during serum incubation. First, we found that inhibition of DNA synthesis by hydroxyurea suppressed the cell death induced by serum (Figure 3A). At concentrations that were non-lethal to WT, 50 and 75 mM hydroxyurea reduced *Fas2Δ/Δ* yeast cell death after 24 h incubation with 50% serum from approximately 78% to 54% and 32%, respectively (Figure S2). The data suggested that synthesis of DNA might lead to a cell death response in the mutant yeast cells. We evaluated whether DNA synthesis was occurring during serum incubation by measuring the DNA content of the WT and *Fas2Δ/Δ* yeast cells. We found that DNA synthesis in both WT and *Fas2Δ/Δ* yeast cells was occurring as the population of G2 cells (4 N) increased over the period of 4 h of incubation compared with initial populations (before shifting to serum) (Figure 3B). The G2 populations of WT cells increased from 7% to 47% at 4 hours, whereas the mutant's G2 populations increased from 13% to 64%. Hence, at 4 h, the G2 population was higher in the mutant cultures compared to the WT (Figure 3B). The addition of 75 mM hydroxyurea suppressed the production of G2 cells in both strains. As shown above (Figure 1), WT cells proliferated in serum media and the accumulation of G2 cells reflects ongoing replication in the daughter and mother cells. In contrast, the *Fas2Δ/Δ* cells failed to produce progenies and cell death was induced. Interestingly, cell death was not significantly affected by the addition of nocodazole, an inhibitor of cell cycle at G2/M phase (Figure S2B), indicating that cell death could only be blocked by impeding early steps in the cell cycle. These data indicated that DNA synthesis was actively occurring in the mutant yeast cells despite the blockade of fatty acid synthesis and support the hypothesis of a metabolic origin of cell death in the mutant yeast.

Cell death of the *Fas2Δ/Δ* strain was triggered by glucose metabolism, suggesting the involvement of an active process. Since suppression of protein synthesis by CHX had been shown to inhibit cell death induced by serum in a *C. albicans* homoserine kinase mutant [26] or induced by fatty acid starvation in *S. cerevisiae* [27], we examined the effect of CHX on cell death in *C. parapsilosis Fas2Δ/Δ* yeast cells. Concentrations of 50 µg/ml of CHX in serum rescued up to 98–100% and 85–92% of *Fas2Δ/Δ* yeast cells from death at 24 h and 48 h of serum incubation, respectively (Figure 3C, S1). These results indicated that cell death induced by serum is linked to protein biosynthesis.

### *Fas2Δ/Δ* yeast cells displayed enhanced metabolic activity resulting in mitochondrial dysfunction

**Figure 4:**
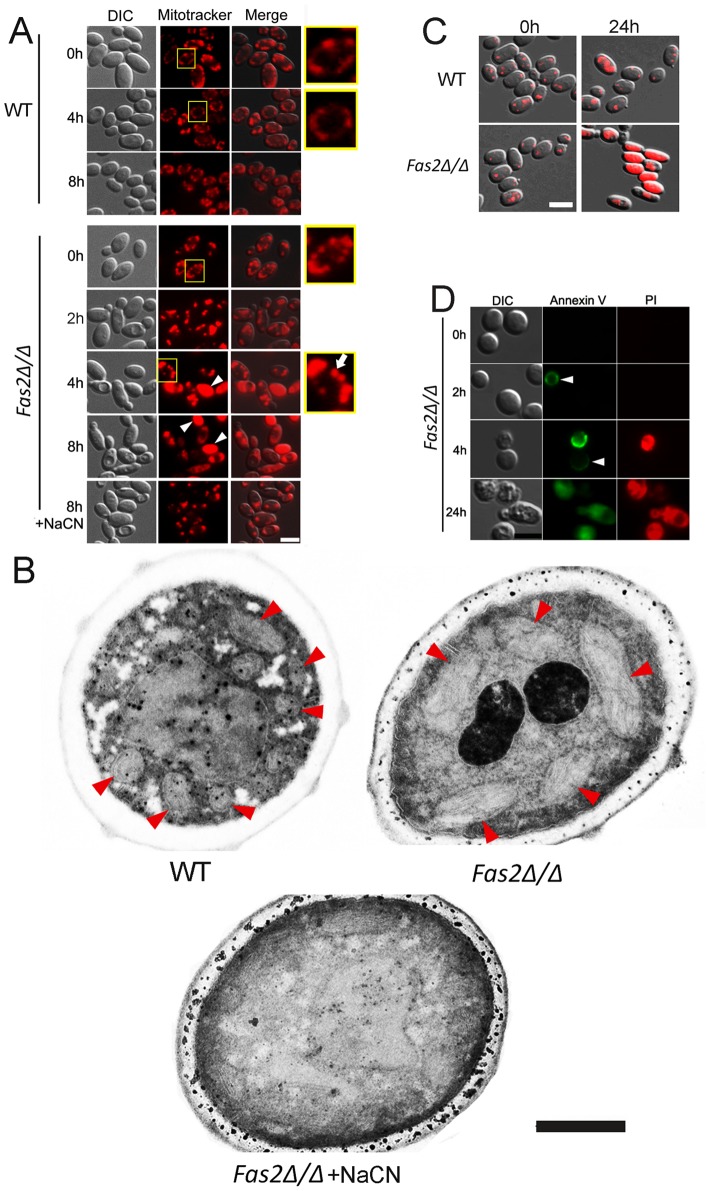
The death of *Fas2Δ/Δ* yeast cells in serum is mediated via mitochondria. A, Evidence of mitochondrial fragmentation after serum treatment in *Fas2Δ/Δ* yeast cells. Serum treated WT and *Fas2Δ/Δ* yeast cells were stained with mitotracker red CM-H_2_XRos to visualize their mitochondrial networks. WT yeast cells maintained normal mitochondrial architecture over time, whereas the fragmentation and swelling occurred in *Fas2Δ/Δ* mitochondria. Addition of cyanide (NaCN) depolarized mitochondria and inhibited ROS accumulation in *Fas2Δ/Δ* strain preventing mitochondrial fragmentation. Arrowheads show the diffusion of mitotracker dye and the arrow indicates a swollen mitochondria. Scale bar: 5 µm. B, Mitochondrial swelling occurs in *Fas2Δ/Δ* yeast cells incubated in serum. Mitochondria of WT and *Fas2Δ/Δ* strains treated with serum were visualized by transmission electron microscopy. *Fas2Δ/Δ* yeast cell mitochondria are dramatically swollen whereas the mitochondrial are architecturally normal in WT yeast. The addition of cyanide prevented mitochondrial swelling in *Fas2Δ/Δ* yeast cells. Red arrow heads indicate mitochondria. Scale bar: 0.5 µm. C, Evidence of metabolic dysfunction of mitochondrial of mutant cells. Mitochondrial function was assessed by FUN-1. Although FUN-1 did not accumulate over time in the WT yeast cells, it significantly accumulated in the *Fas2Δ/Δ* yeast cells. Scale bar: 5 µm. D, Serum treatment induced cell death in *Fas2Δ/Δ* yeast cells. Green fluorescence indicated staining with annexin V, whereas red fluorescence showed labeling with propidium iodine (PI). Scale bar: 5 µm. The experiments shown were performed at least twice with reproducible data.

Our results indicated that *C. parapsilosis Fas2Δ/Δ* cell death in serum and glucose containing media is an active process and that metabolic activity might be intimately involved in cell death. Therefore, we hypothesized that the presence of glucose activates *Fas2Δ/Δ* yeast cell metabolic activity, which triggers DNA and protein synthesis. We postulated that these energy-consuming processes were related to mitochondrial functions. Thus, we first examined yeast cell mitochondrial morphology using mitotracker red CM-H_2_XRos, which is a reduced, non-fluorescent form that fluoresces upon oxidation. We found that WT and *Fas2Δ/Δ* yeast cells incubated in medium with fatty acids (time 0 h) displayed similar mitochondrial morphologies in which mitochondria localized in proximity to cell membranes (Figure 4A). The mitochondrial morphology of WT yeast cells cultivated in serum for 4 h and 8 h was unchanged, whereas mitochondria of >90% of *Fas2Δ/Δ* yeast cells were fragmented after 2 h and significantly swollen within 4 h (Figure 4A). Additionally, portions of the *Fas2Δ/Δ* yeast cells were not labeled by the dye, indicating significant mitochondrial damage. Other *Fas2Δ/Δ* yeast cells accumulated large amounts of mitotracker red and the number of yeast cells accumulating the dye increased over time. Since mitotracker red is also used to measure ROS accumulation, the bright fluorescence in the *Fas2Δ/Δ* yeast cells might be consistent with high levels of ROS. Notably, treatment of *Fas2Δ/Δ* yeast cells with cyanide prevented the accumulation of the dye, albeit mitochondrial morphology was also affected (Figure 4A). These results indicated that treatment of the *Fas2Δ/Δ* yeast cells with serum or YPD resulted in mitochondrial dysfunction.

The enhanced staining of *Fas2Δ/Δ* yeast cells in serum suggested that the cells had swollen mitochondria. Thus, we examined the mitochondrial morphology of WT and *Fas2Δ/Δ* yeast cells by electron microscopy and found that the mitochondria of the mutant cells were significantly larger (Figure 4B) compared with WT. Thus, swellings of mitochondria after serum treatment further suggested the dysfunction of mitochondria in the *Fas2Δ/Δ* yeast cells.

Overproduction of ROS can cause mitochondrial injury resulting in diminished mitochondrial activity. Thus, we examined mitochondrial activity by staining cells with FUN1. In metabolically active yeast cells, FUN-1 is transported to the vacuole and converted into a cylindrical intravacuolar structure [28]. This process is mitochondrial ATP dependent. We have found that treatment of the *Fas2Δ/Δ* yeast cells for 24 h with serum leads to the diffusion of FUN-1 (Figure 4C), suggesting a significant impairment in mitochondrial function.

To further document cell death in the *Fas2Δ/Δ* yeast cells after serum treatment, we assayed cell viability using annexin V and PI as a markers for cell death. Treatment of *Fas2Δ/Δ* yeast cells with 50% serum for 2 or 4 h resulted in annexin V staining of 8.5% and 23.5% cells, respectively (Figure 4D). We also found that *Fas2Δ/Δ* yeast cells were co-stained with annexin V and PI after 24 h incubation (Figure 4D). Hence, the data indicate that serum induced cell death in *Fas2Δ/Δ* yeast cells.

We showed that mitochondrial dysfunction in the presence of glucose in a medium without fatty acids resulted in the death of *Fas2Δ/Δ* yeast cells. Cell death was rescued by cyanide or azide, which target complex IV in the electron transport chain in the mitochondria (see below). We further investigated the role of the electron transport chain in *Fas2Δ/Δ* cell death by testing the effects of various inhibitors of the electron transport chain as well as other cellular pathways on cell viability (summarized in table 1). Antimycin A and FCCP, which inhibit complex III and lead to uncoupling of the electron transport chain with oxidative phosphorylation, respectively, rescued the *Fas2Δ/Δ* yeast cells from the otherwise toxic effects of serum and YNB with glucose, although their effects were less potent than seen with cyanide, azide and CHX. In contrast, rotenone and oligomycin, which respectively target complex I and V, did not prevent the death of *Fas2Δ/Δ* yeast cells. Additionally, inhibition of other cellular pathways, such as autophagy or ceramide synthesis, did not rescue the mutant yeast cells. These results supported our conclusion that cell death was mediated by mitochondria and highlighted the role of the electron transport chain in this process.

**Table 1:** Summary of the effect of pharmacological drugs on *Fas2Δ/Δ* cell viability.

Inhibitor	Target	% inhibition of cell death	
Mitochondrial function		Serum	YNB with glucose
Rotenone (10 µg/ml)	Complex I	N.I.	N.I.
Antimycin A (2.5 µg/ml)	Complex III	>50%	>75%
Cyanide (1 mM)	Complex IV	>98%	>98%
Azide (2.5 mM)	Complex IV	>95%	>95%
Oligomycin (10 µg/ml)	Complex V	N.I.	N.I.
FCCP (1 mM)	Uncoupler	>50%	>75%
**Other pathways**			
Cycloheximide (50 µg/ml)	Protein synthesis	>94%	>94%
Hydroxyurea (75 mM)	DNA synthesis	>70%	>70%
Nocodazole (50 µg/ml)	Mitosis	<8%	N.I.
Wortmannin (100 µM)	Autophagy	N.I.	N.I.
Fumonisin b1(1.38 mM)	Ceramide synthase	N.I.	N.I.
aCerulenin (10 µg/ml)	Fatty acid synthase	N.I.	N.I.

N.I., no inhibition; parenthesis, concentration tested.

Serum: 50% fetal bovine serum diluted with water; YNB, yeast nitrogen base with amino acids.

a negative control.

### Mitochondrial ROS accumulation in *Fas2Δ/Δ* yeast cells is responsible for cell death

**Figure 5:**
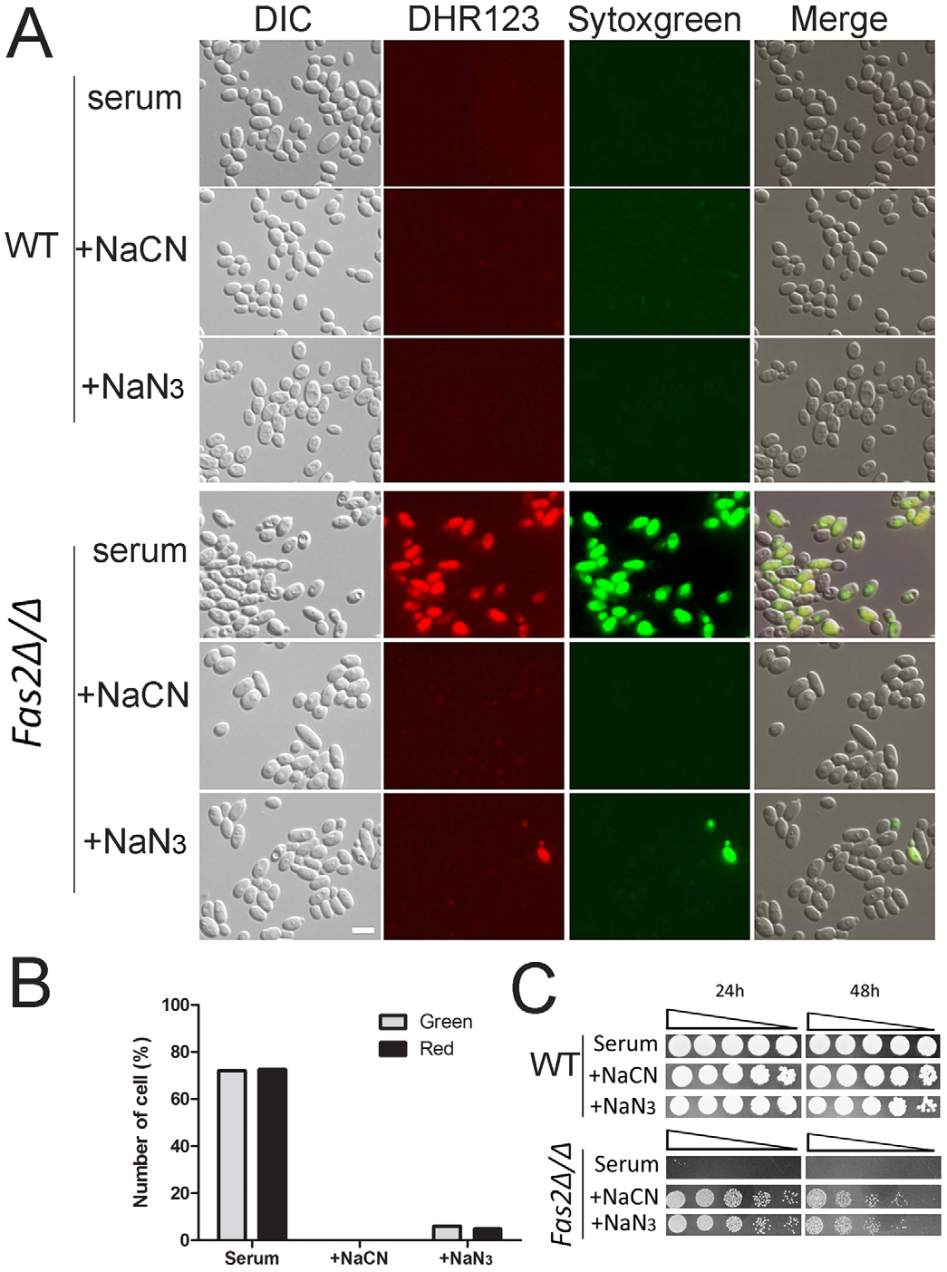
Cell death results from overproduction of mitochondrial ROS. A, Accumulation of mitochondrial ROS in *Fas2Δ/Δ* yeast cells after serum treatment. WT and *Fas2Δ/Δ* yeast cells were treated with 50% serum with or without 1 mM sodium cyanide (NaCN) or 2.5 mM sodium azide (NaN_2_) for 24 h at 30°C. Yeast cells were stained with dihydrorhodamine 123 and Sytogreen to visualize ROS and dead cells, respectively. Scale bar: 5 µm. B, Accumulation of ROS coincided with the death of *Fas2Δ/Δ* yeast cells. The numbers of red (ROS positive) and green (Sytogreen positive) were assessed for *Fas2Δ/Δ* yeast cells treated for 12 h with serum in the presence or absence of cyanide or azide. C, Inhibition of mitochondrial respiration rescued death of *Fas2Δ/Δ* yeast cells. Spot assay on YPDT40 of yeast cells incubated with serum in the presence or absence of 1 mM sodium cyanide or 2.5 mM sodium azide. Experiments were performed three times with reproducible results.

We showed that serum treatment resulted in dysfunction of mitochondria and accumulation of ROS. We further assessed the effects of ROS by staining serum treated yeast cells with DHR123 as a ROS accumulation marker and Sytoxgreen as an indicator of cell death. Although the dyes did not label WT yeast cells cultivated in serum, the *Fas2Δ/Δ* yeast cells displayed significant accumulations of DHR123 (Figure 5A). These mutant cells labeled with DHR123 also exhibited the green fluorescence of sytoxgreen indicating cell death. To confirm that ROS accumulation was a result of the dysfunction of mitochondria after serum treatment in the *Fas2Δ/Δ* yeast cells, we used cyanide and azide to block the electron transport chain. Treatment with cyanide or azide significantly suppressed the formation of ROS in the *Fas2Δ/Δ* yeast cells and enhanced cell viability (Figure 5A, B, and C). Also, the addition of the anti-oxidant N-acetyl cysteine (NAC) partially reduced mutant cell death (Figure S1B, S2B). In sum, these data demonstrated that the death of *Fas2Δ/Δ* yeast cells in serum was subsequent to mitochondrial ROS production.

### Cell death is partially dependent on mitochondrial DNA

**Figure 6:**
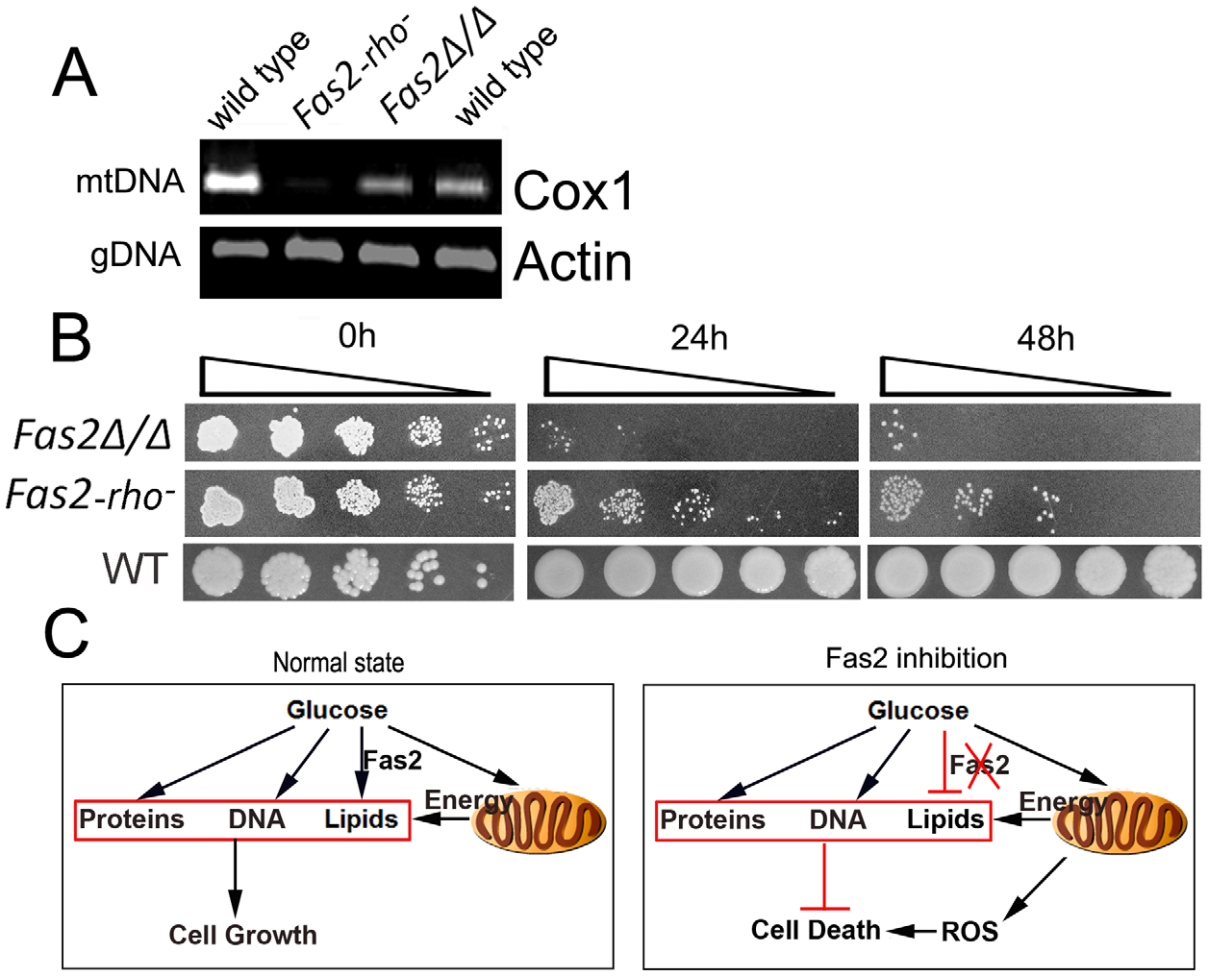
Cell death is dependent on mtDNA. A, mitochondrial DNA and genomic DNA were evaluated by PCR from total DNA isolated from the indicated strains. Mitochondrial DNA content was assessed using the subunit I of cytochrome c oxidase gene (Cox1). Genomic DNA content was determined using the actin gene (Actin). B, Deletion of mtDNA protected *Fas2Δ/Δ* yeast cells from death induced by culture in serum. WT, *Fas2Δ/Δ* and *Fas2Δ/Δ* with partially deleted mtDNA (*Fas2-rho^−^*) were incubated with 50% serum for 24 and 48 h. Aliquots were serially diluted and WT yeast were spotted onto YPD whereas the *Fas2Δ/Δ* mutants were inoculated onto YPDT40. Experiments were performed twice with similar results. C, A proposed model of yeast cell sensitivity to glucose in the media in the setting of fatty acid inhibition.

Our results pointed to mitochondrial dysfunction as the primary factor in the death of *Fas2Δ/Δ* yeast cells in the presence of serum or glucose. We found that cyanide or azide treatment arrested the lethal affects of glucose exposure. Several enzymes and proteins in the electron transport chain, such as cytochrome c oxidase, are encoded by mtDNA and are inhibited by cyanide and azide. Thus, we hypothesized that deletion of mtDNA might reduce *Fas2Δ/Δ* cell death induced by serum or YPD. To this end, we deleted the mtDNA of the *Fas2Δ/Δ* yeast cells (Figure 6A). The survival of *Fas2Δ/Δ* yeast cells defective in mtDNA (Rho negative) was examined after serum treatment. As predicted, we observed that deletion of mtDNA significantly decreased the death of *Fas2Δ/Δ* yeast cells in our media (Figure 6B). Nevertheless, loss of mtDNA did not completely protect cell death. Thus, the data indicate that mtDNA was partially involved in the cell death response in the *Fas2Δ/Δ* strain.

### Fas2 is important for serum stress in the blood stream

**Figure 7:**
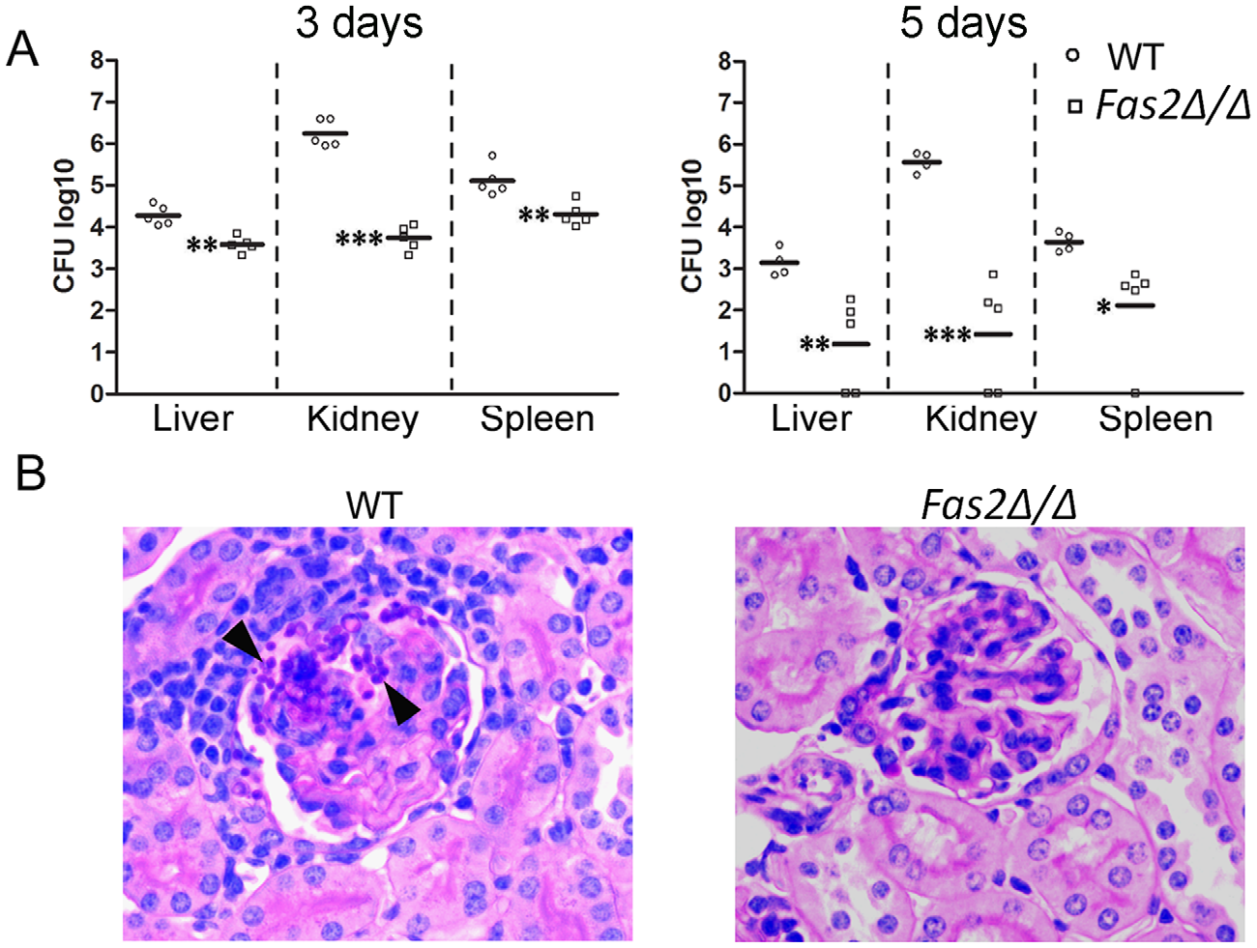
*Fas2Δ/Δ* yeast cells are attenuated in vivo. Intravenous infection of A/J mice with WT and *Fas2Δ/Δ* yeasts (C, D). A, shown are the CFUs from kidneys, spleens, and livers 3 and 5 days intravenous infection. Each symbol represents 1 mouse. *P≤0.05; **P≤0.01; ***P≤0.001, (Newman-Keuls). B, images shown are representative histological sections of kidneys 5 days after intravenous infection with WT and *Fas2Δ/Δ* strain, respectively. Arrowheads indicate aggregates of yeast cells in WT infected tissues. Initial magnification ×400.

Since the *Fas2Δ/Δ* yeast cells were hypersensitive to serum and specific serum components, we examined the virulence of the yeast cells in vivo by injecting the yeast cells intravenously into mice. Significantly fewer *Fas2Δ/Δ* yeast cells compared to WT yeast cells were detected in all organs (Figure 7A). At day 3 after infection, there was a log reduction in *Fas2Δ/Δ* CFU compared to mice infected with WT in the livers and spleens and a 2.5 log reduction in the kidneys. At day 5, there was at least a 2 log reduction in *Fas2Δ/Δ* CFU relative to WT in all organs. Moreover, *Fas2Δ/Δ* yeast cells were undetectable in several mice. The kidney is the non-lymphoid organ where blood is filtered. At day 5 after infection, WT yeast cells were clearly visible within tubules and glomeruli and multifocal, widespread granulomatous and pyogranulomatous inflammation was present (Figure 7B). In contrast, we were unable to identify any *Fas2Δ/Δ* yeast cells in the PAS stained section by light microscopy and the kidneys were histologically normal. Hence, the *Fas2Δ/Δ* yeast cells were attenuated in an intravenous infection model.

## Discussion

Essential genes are often the preferred pharmacological targets in the treatment of infectious diseases. Auxotrophic yeast mutants are, therefore, important models for studying the impact of essential genes. Several auxotrophic mutants of *S. cerevisiae* die in media lacking appropriate nutrients to support their growth [27], [29]. Such studies have revealed that rather than arresting their growth in the absence of required nutrients, the metabolic processes of *S. cerevisiae* cells lacking certain essential genes (e.g. fatty acid synthesis genes) can be functioning in an uncontrolled and unbalanced manner, resulting in cell death [30], [31]. We have observed that the deletion of *FAS2* genes in *C. parapsilosis* results in a fatty acid auxotrophic mutant, which is hypersensitive to serum and glucose containing media. Although, *C. parapsilosis Fas2Δ/Δ* yeast cells are not able to replicate in media without exogenous fatty acids, the growth of these mutant cells are similar to WT in glucose containing media supplemented with appropriate fatty acids [3]. Interestingly, serum contains fatty acids and lipids, thus we initially suspected that serum would support the growth of *Fas2Δ/Δ* yeast cells, meaning that inhibition of Fas2 could be bypassed by serum fatty acids as observed in bacteria [8]. We previously described that Fas2 mutant growth is rescued by saturated fatty acids, but not unsaturated ones, and the growth of *Fas2Δ/Δ* yeast cells is also dependent on amount of saturated fatty acids [3]. Furthermore, the amount of saturated fatty acids in serum is low and mostly sequestered by albumin. Thus, we proposed that the limited levels of saturated fatty acids in serum could not promote the growth of *Fas2Δ/Δ* yeast cells. In contrast, we found that *Fas2Δ/Δ* yeast cells rapidly die in serum. Serum contains approximately 0.1% glucose and our results show that glucose is one of the key factors for triggering the death response of *Fas2Δ/Δ* yeast cells.

Glucose is a preferred carbon source for yeast growth. Thus, it is unexpected that glucose is a toxic factor to the *Fas2Δ/Δ* yeast cells. Although the mechanism for glucose toxicity in the *Fas2Δ/Δ* remains unclear, our results suggest that uncontrolled metabolism of glucose by the mutant yeast cells results in unbalanced synthesis of cellular contents, such as DNA or proteins, which could trigger a cell death response. In addition, the presence of glucose might facilitate energy production for energy-requiring cell death pathways such as apoptosis [32]. Indeed, we have found that replacement of glucose with other non-favorable carbons, such as glycerol, or ethanol, dramatically increases the viability of *Fas2Δ/Δ* cells, supporting the notion that cell death is induced by energy-rich compounds. Interestingly, metabolism of glucose in the absence of other nutrients does not sensitize the *Fas2Δ/Δ* yeast cells [33]. Thus, the induction of cell death by glucose is not purely due to the accumulation of toxic intermediates from glucose metabolism. Instead, our evidence supports the notion that glucose triggers cell cycle progression, which results in asymmetric accumulation of cellular contents in the auxotrophic yeast cells. Survival of auxotrophic yeast cells is also dependent on nutrient availability, and we have determined that fermentable carbon sources, such as glucose, induce rapid loss of viability compared with non-fermentable carbon sources in leucine or uracil auxotrophic yeasts [30], [31]. Our findings and evidence in the literature [31] indicate that auxotrophic yeast cells do not enter a resting state when rich nutrients are available and the survival of auxotrophic mutants is dependent on specific growth conditions.

We have found that the time to death of the *Fas2Δ/Δ* yeast cells incubated in serum or glucose containing media corresponds with the dysregulation of cell responses, such as bud emergence or formation of vesicles within a large cytoplasmic vacuole (Figure S3). Although the latter suggests that there could be an autophagic cell death of *Fas2Δ/Δ* yeast cells, inhibition of autophagy with wortmannin does not rescue the yeast (Table 1). Besides, inhibition of fatty acid synthase in mammalian cells leads to the accumulation of malonyl-CoA and up-regulation of ceramide [34]. Also, *Fas2Δ/Δ* yeast cell death is not prevented by the ceramide synthase inhibitor fumonisin b1, which suggests that yeast cell death is not subsequent to ceramide accumulation. We have also ruled out toxicity due to the accumulation of malonyl-CoA as the inhibitor of beta-oxidation leading to cell death, since 1% glucose is sufficient to inhibit this process in yeast [35], [36]. The fact that the other carbon sources tested such as ethanol are non-toxic to the mutant yeast also suggests that the accumulation of malonyl-CoA would not be lethal. Thus, the death of *Fas2Δ/Δ* yeast cells triggered by serum or glucose containing media is specifically initiated by glucose metabolism.

Intriguingly, we have found that inhibition of protein or DNA synthesis suppresses the cell death response in the mutant cells. The data suggest that ongoing processes in the *Fas2Δ/Δ* yeast cells cause cell death and that the mutant cells do not enter a quiescent state in serum or glucose containing media. The presence of glucose in the media might induce a rapid expression of genes involved in cellular division. Therefore, we propose that the mutant cells die because of proteotoxic and/or replication stress. Nevertheless, the mechanism of the death of *Fas2Δ/Δ* yeast cells caused by the accumulation of proteins and DNA is unclear. At this point, we can only speculate that an asymmetric accumulation of cellular contents in the non-dividing mutant causes the cell death.

Mitochondria are the major site of ATP production. Additionally, these organelles regulate cell death programs when yeast cells encounter stress conditions that potentially affect certain cell populations, such as aged or hypoxic cells [37]. We have found that *Fas2Δ/Δ* cell death is induced by up-regulation of mitochondrial ROS. ROS overproduction triggers disruption of yeast cell mitochondrial networks and severely damages the mitochondria. These events are blocked by pre-treatment of the cells with electron transport chain inhibitors such as cyanide or azide, which inhibits cytochrome c oxidase preventing the generation of ATP in the mitochondria, indicating that cell death is energy-dependent. We propose that glucose in the media induces the production of mitochondrial ROS in response to the accumulation of cellular components in the non-dividing mutant yeast cells. Indeed, glucose has been known to stimulate ROS production and apoptosis in *S. cereviseae* [38]. Nonetheless, it is unclear how glucose would induce production of ROS. One possibility is that glucose triggers cell cycle progression and inhibits quiescence in the non-dividing yeast cells that could lead to ROS accumulation and cell death as observed in chronologically aged yeast cells [37]–[39]. Moreover, the electron transport chain is responsible for ROS production [40], [41]. Thus, disruption of mtDNA might abolish ROS accumulation as observed in *S. cerevisiae* [21]. Indeed, *Fas2Δ/Δ* yeast cell death is partially suppressed by deletion of mtDNA. Thus, functional mitochondria are key factors for determining cell death of *Fas2Δ/Δ* yeast cells. Thus, our data highlight a link between the fatty acid synthesis pathway and mitochondrial function.

The dissemination of *C. parapsilosis* yeast relies on its ability to cope with serum stress. Fas2 impacts yeast cell survival in vivo as the *Fas2Δ/Δ* cells are significantly more effectively eliminated from livers, spleens and kidneys after intravenous injection compared to WT yeast cells. Given that the yeast cells are bathed in serum factors during dissemination and within tissues, the eradication of the *Fas2Δ/Δ* yeast cells may in part be attributed to the toxic effects of the serum factors. This validates and provides a mechanism to explain our prior report where *Fas2Δ/Δ* yeast cells were less able to disseminate after intraperitoneal inoculation compared to WT yeast cells [3]. Serum lipids can subvert the inhibition of certain bacterial fatty acid synthases [8], [42]. We show that mixtures of fatty acids and lipids extracted from serum supported *Fas2Δ/Δ* yeast cell replication and growth, indicating that serum fatty acids/lipids can potentially overcome the otherwise lethal effects of serum. However, these conditions are different from those found *in vivo* where, for instance, saturated fatty acids are sequestered by albumin. Interestingly, we have determined that approximately 40% of *Fas2Δ/Δ* yeast cells incubated in serum are able to produce single, non-detached buds, which are non-viable within hours of inoculation into serum. We propose that the bud emergence in 40% of the mother cells could be the results of successful utilization of serum lipids or stored lipids (e.g. lipid droplets) by the G2-phase cells as the cells are non-synchronized before shifting to serum. The presence of glucose could facilitate the progression of cell cycle in a portion of *Fas2Δ/Δ* yeast cells. However, the mutant cells lack essential fatty acids for cell membrane synthesis to complete their cell cycle, resulting in the death response. Hence, during infection there is a complex balance in the *Fas2Δ/Δ* yeast cells between the toxic effects of glucose and other factors and the utilization of host lipids/fatty acids.

Fatty acid synthesis is known as an essential pathway from bacteria to humans. However, few organisms have evolved to survive without requiring the functions of this pathway. For example, *Malassezia* species are human commensal yeasts that lack the enzymes of the fatty acid synthesis pathway [43]. In order to grow, the yeasts deploy a repertoire of lipolytic enzymes to acquire fatty acids from host lipids [44]. We have also shown that the *Fas2Δ/Δ* yeast cells are able to grow on media supplemented with lipids, suggesting that inhibition of Fas2 alone might not be sufficient in lipid-rich host niches [45]. However, this inhibition is lethal in serum media. Thus, fatty acid synthase might be essential for yeasts to survive and disseminate in a complex in vivo milieu.

From the above results, a model of yeast cell death in response to glucose in the media when fatty acid synthesis inhibited is proposed (Figure 6C). In conditions where fatty acids synthesis is intact, glucose is used for fatty acid synthesis and then lipid species for cell membrane buildup, thus enabling the generation of new progenies. When fatty acid synthesis is inhibited, the utilization of glucose is not suppressed leading to the uncontrolled synthesis of cellular components such as proteins and nucleic acids. The asymmetric accumulation of cellular contents stresses the yeast cells and induces cell death. Mitochondria seem to play an essential role in the cell death response by up-regulation of ROS in the *Fas2Δ/Δ* yeast cells. Hence, our data shows that Fas2 regulates fatty acid synthesis from glucose metabolism, which is important for cellular homeostasis in dividing cells. Furthermore, inhibition of Fas2 is fungicidal to yeasts in serum and rich media as a result of mitochondrial dysfunction. Therefore, yeast fatty acid synthesis is a promising target for antifungal development.

## Supporting Information

Figure S1 **Analysis of cell death of yeast cells in YNB.** A) WT and *Fas2Δ/Δ* yeast cells were grown for 24 h in YNB media supplemented with the indicated carbon source. B) *Fas2Δ/Δ* yeast cells were grown for 36 h in the presence or absence of the indicated concentration of glucose, nocodazole or N-acetyl cysteine (NAC). Yeast cells were stained with Sytoxgreen and counted by FACS. The percentages of live and dead cells were calculated from >25,000 cells. Experiments were performed at least twice with duplicates and similar results were obtained.
(PDF)

Figure S2 **Analysis of cell death of yeast cells in serum.** A) WT and *Fas2Δ/Δ* yeast cells were cultured in 50% serum with or without hydroxyurea (HU) or cycloheximide (CHX). B) *Fas2Δ/Δ* yeast cells were grown in serum with or without nocodazole or N-acetyl cysteine (NAC). *Fas2Δ/Δ* yeast cells were also incubated in the >10 kDa serum fraction with or without glucose and contrasted to the <10 kDa fraction. Yeast cells were recovered after 24 h incubation at 30°C, stained with Sytoxgreen and counted by FACS. The percentages of live and dead cells were calculated from >30,000 cells. Experiments were performed at least twice in duplicates with similar results.
(PDF)

Figure S3 **Incubation of** ***Fas2Δ/Δ*** **yeast cells with serum induced vacuolation (indicated by arrowheads).** This phenotype was inhibited with 50 µg/ml CHX. Scale bar: 5 µm. Experiments were performed twice with similar results.
(PDF)
