# Supplementary figures and images for: Inhibition of Candida parapsilosis Fatty Acid Synthase (Fas2) Induces Mitochondrial Cell Death in Serum
Source: PLoS Pathog. 2012 Aug 30;8(8):e1002879. doi: 10.1371/journal.ppat.1002879 (PMC3431346; doi:10.1371/journal.ppat.1002879)

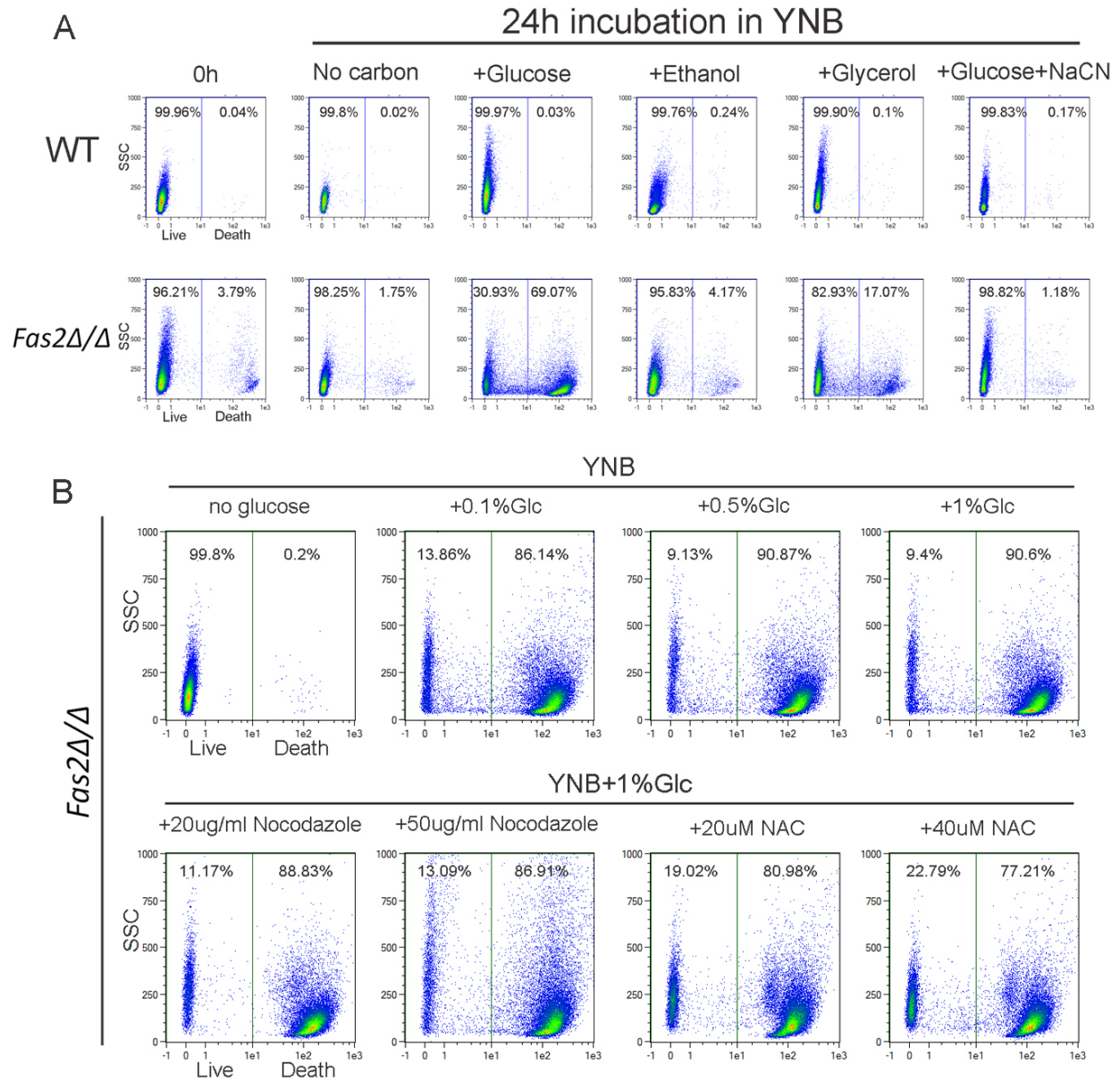

**Figure S1.**

Supplement: Figure S1 — Analysis of cell death of yeast cells in YNB. A) WT and Fas2Δ/Δ yeast cells were grown for 24 h in YNB media supplemented with the indicated carbon source. B) Fas2Δ/Δ yeast cells were grown for 36 h in the presence or absence of the indicated concentration of glucose, nocodazole or N-acetyl cysteine (NAC). Yeast cells were stained with Sytoxgreen and counted by FACS. The percentages of live and dead cells were calculated from >25,000 cells. Experiments were performed at least twice with duplicates and similar results were obtained. (PDF) [file ppat.1002879.s001.pdf]

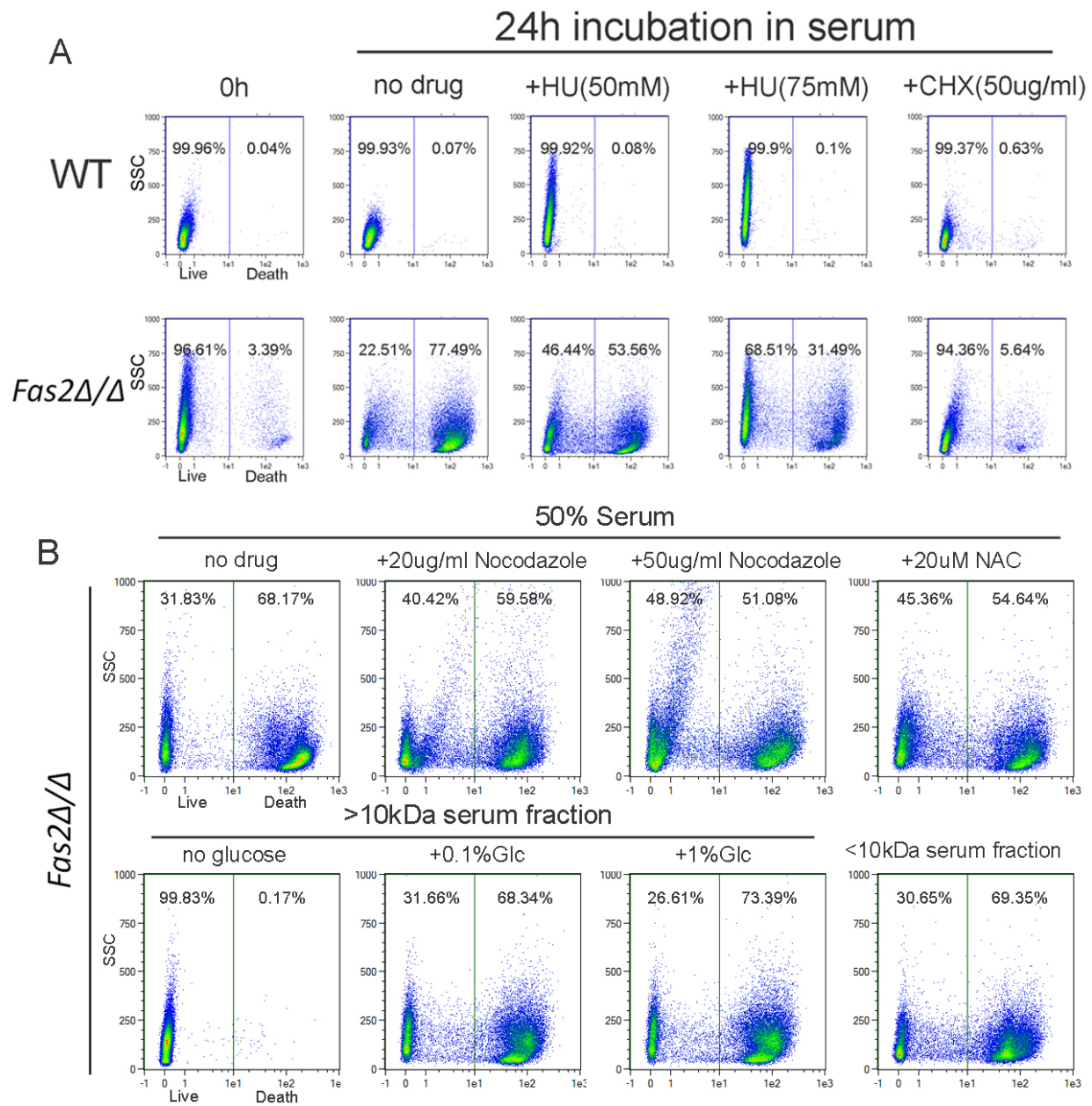

**Figure S2.**

Supplement: Figure S2 — Analysis of cell death of yeast cells in serum. A) WT and Fas2Δ/Δ yeast cells were cultured in 50% serum with or without hydroxyurea (HU) or cycloheximide (CHX). B) Fas2Δ/Δ yeast cells were grown in serum with or without nocodazole or N-acetyl cysteine (NAC). Fas2Δ/Δ yeast cells were also incubated in the >10 kDa serum fraction with or without glucose and contrasted to the <10 kDa fraction. Yeast cells were recovered after 24 h incubation at 30°C, stained with Sytoxgreen and counted by FACS. The percentages of live and dead cells were calculated from >30,000 cells. Experiments were performed at least twice in duplicates with similar results. (PDF) [file ppat.1002879.s002.pdf]

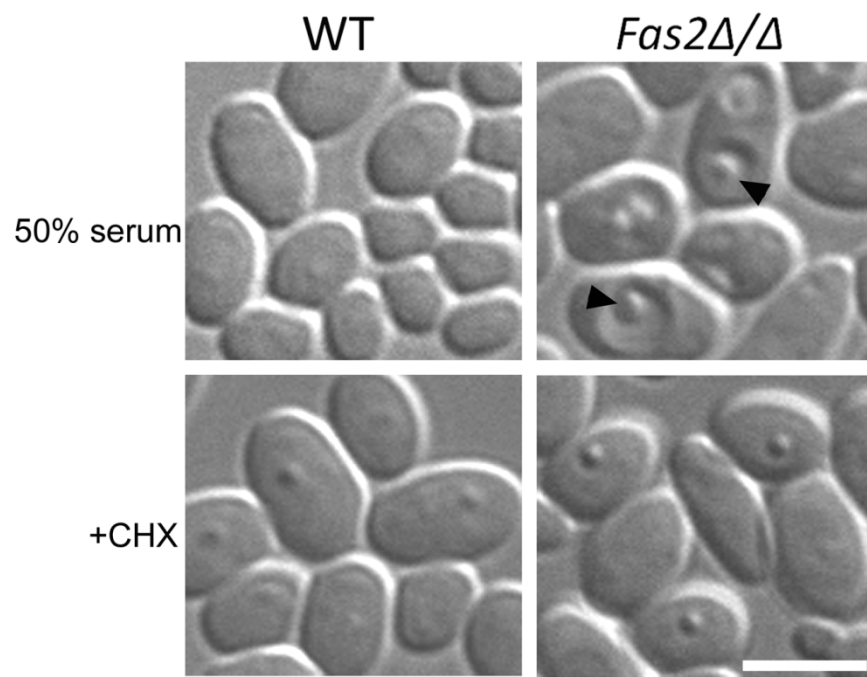

**Figure S3.**

Supplement: Figure S3 — Incubation of Fas2Δ/Δ yeast cells with serum induced vacuolation (indicated by arrowheads). This phenotype was inhibited with 50 µg/ml CHX. Scale bar: 5 µm. Experiments were performed twice with similar results. (PDF) [file ppat.1002879.s003.pdf]
